# Application and Optimization of *relE* as a Negative Selection Marker for Making Definitive Genetic Constructs in Uropathogenic *Escherichia coli*

**DOI:** 10.3390/pathogens5010009

**Published:** 2016-01-18

**Authors:** Varnica Khetrapal, Kurosh S. Mehershahi, Siyi Chen, Swaine L. Chen

**Affiliations:** 1 Department of Medicine, Yong Loo Lin School of Medicine, National University of Singapore, 1E Kent Ridge Road, NUHS Tower Block, Level 10, Singapore 119074, Singapore; khetrapalv@gis.a-star.edu.sg (V.K.); mehershahiks@gis.a-star.edu.sg (K.S.M.); sychen@gis.a-star.edu.sg (S.C.); 2 Genome Institute of Singapore, Infectious Diseases Group, 60 Biopolis street, Genome, #02-01, Singapore 138672, Singapore

**Keywords:** negative selection, UTI89, FimH

## Abstract

Studies of Uropathogenic *Escherichia coli* (UPEC) pathogenesis have relied heavily on genetic manipulation to understand virulence factors. We applied a recently reported positive-negative selection system to create a series of unmarked, scarless FimH mutants that show identical phenotypes to previously reported marked FimH mutants; these are now improved versions useful for definitive assignment of phenotypes to FimH mutations. We also increased the efficiency of this system by designing new primer sites, which should further improve the efficiency and convenience of using negative selection in UTI89, other UPEC, and other Enterobacteriaceae.

## 1. Introduction

Urinary tract infections (UTIs) are a major problem in women worldwide, with uropathogenic *Escherichia coli* (UPEC) causing 74% of community-acquired and 65% of nosocomial infections [1]. Several UPEC strains, including *Escherichia coli* (*E. coli*) UTI89, have been shown to form intracellular bacterial communities (IBCs) in mice and in humans [2,3,4]; IBCs may be responsible for recurrent UTIs in mice [5]. Type 1 pili, encoded by the *fim* operon in nearly all *E. coli*, is the major virulence factor for UPEC during UTI [6,7]. FimH is the mannose-binding adhesin found at the tip of type1 pili which mediates binding to mannosylated proteins on bladder epithelial cells [8,9,10,11], facilitating colonization and invasion of cells *in vitro* [12,13] and *in vivo* [6,14] and enabling IBC formation [14,15,16].

Previous FimH studies have used isogenic strains carrying mutant *fimH* alleles on plasmids [17] or on the chromosome [14,16]. Chromosomal strains, in particular, have the advantages of native copy number and chromosomal context, and therefore native transcriptional regulation. However, manipulating the chromosome of clinical *E. coli* isolates such as UTI89 is more challenging than in lab-adapted strains, for which most cloning systems have been developed. This results in strains that are either marked [14,16] or carry residual cloning scars [18]. Formally, from a genetic point of view, creation of unmarked, scarless mutations in genes such as *fimH* would enable the strongest possible assignment of phenotype changes to allelic differences.

We have recently published a novel negative selection system to facilitate chromosomal manipulation in UTI89 [19]. This system consists of a toxic gene (*relE*) expressed from a tightly controlled promoter, such as the rhamonse-inducible P_*rhaB*_ promoter. Under normal growth conditions, the P_*rhaB*_ promoter is not active, no *relE* is transcribed, and cells grow normally. However, under rhamnose induction, the production of *relE* transcript results in the production of the RelE toxin (an mRNAse), which then stops cell growth. Thus, under restrictive conditions, only cells that do not contain a functional negative selection cassette are able to grow. This negative selection cassette is combined with the kanamycin resistance gene to create a dual positive-negative selection cassette, which was used in a simple two-step procedure to create unmarked, scarless mutations in *fimH* in its native chromosomal context. We verified that our new unmarked strains show no *in vitro* or *in vivo* differences from their corresponding marked strains, validating that the previously reported phenotypes are indeed due to FimH and that the negative selection system generates no artifactual phenotypes in a large set of independent cloning steps. Furthermore, we discovered that most undesired mutants during recombineering (which are subsequently screened out during routine strain verification) using this system were due to recombination at short homology sites in the selection cassette; we eliminated this with newly designed template priming sites. The strains we have created here will be useful in the future for the definitive assignment of phenotypes to FimH mutations, while our modified negative selection protocol will increase the efficiency and convenience of creating unmarked, scarless mutations in UTI89 and other clinical isolates of *E. coli*.

## 2. Results and Discussion

### 2.1. Creation of an Isogenic Series of Definitive (Unmarked, Scarless) FimH Mutants

**Figure 1:**
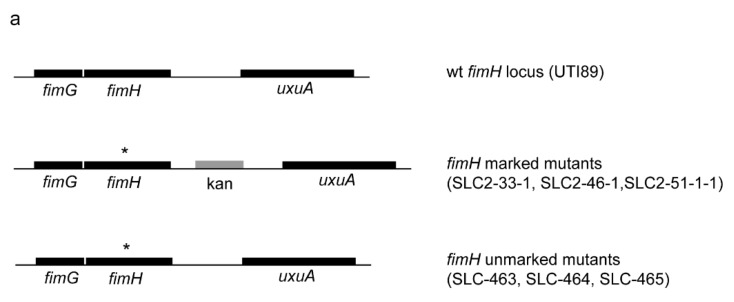

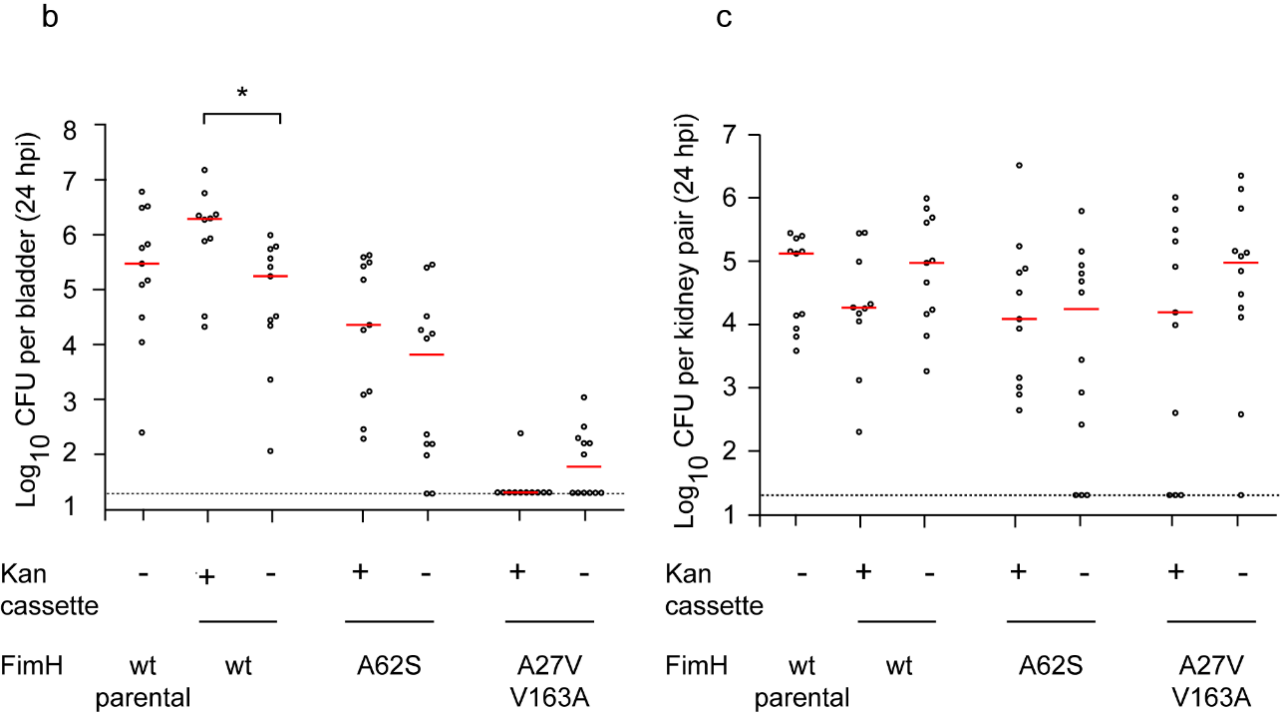
Creation and validation of unmarked FimH mutant strains. (**a**) Genetic organization of the *fimH* locus in UTI89 and in marked and unmarked FimH mutant strains. The top diagram depicts the native, wild-type locus of *fimH* in UTI89 with adjacent genes. The middle diagram depicts the genetic organization surrounding the mutated *fimH* gene (denoted by *) in marked strains reported in [14], indicating the location of the kanamycin selection marker. The bottom diagram depicts the genetic organization surrounding the mutated *fimH* gene (denoted by *) in the strains created in this study. (**b**,**c**) Bladder (**b**) and kidney (**c**) titers from *in vivo* murine infections at 24 hpi. FimH mutations are shown on *x*-axis. Strains carrying the marker (kanamycin cassette) are indicated. *y*-axis plots the logarithm (base 10) of the bacterial CFU measured in the designated organ at 24 hpi. Red horizontal bars indicate medians. Dotted line indicates limit of detection. * *p* = 0.01, two tailed Mann-Whitney test. Data from two independent experiments with five to seven mice in each experiment for each strain shown.

We used the kan-P_*rhaB*_-*relE* positive-negative selection cassette [19] to recreate an unmarked, isogenic series of previously characterized FimH mutants (Figure 1a and Table S1) [14]. These newly created strains no longer carry a linked antibiotic resistance cassette, and therefore are theoretically definitive genetic constructs for isolating phenotypes due to the corresponding FimH mutation. These new unmarked strains therefore also can be utilized in identical assays as the parental UTI89, including transformation with kanamycin-resistant plasmids or the use of kanamycin in subsequent chromosomal manipulations.

### 2.2. Definitive FimH Constructs Have No Artifactual Phenotypes

To validate that the new unmarked *fimH* mutant strains indeed recapitulate previously reported phenotypes, we tested them for *in vitro* type 1 pilus function using guinea pig red blood cell agglutination. We saw no difference between any of the eight pairs of marked and unmarked strains (Table S2). We then tested three alleles in an *in vivo* murine model of urinary tract infection. Marked versions of the wild-type, A62S, and A27V/V163A FimH alleles previously were shown to have no defect, a 1.5-log defect, or a 4–5 log defect, respectively, at 24 hpi in the bladder [14]. We saw the same phenotypes with the new unmarked strains (Figure 1b), and also verified that the reconstructed, unmarked wild-type FimH strain was not significantly different from the unmodified, parental UTI89. We also saw no difference among matched strains in kidney infection titers (Figure 1c). Therefore, use of a combined positive-negative selection cassette in UTI89 is indeed an effective method for creating definitive genetic constructs. This system is applicable in other Enterobacterial strains, including other UPEC, *E. coli*, and Salmonella, and should also be useful for definitive genetic studies in these organisms as well.

### 2.3. Redesigned Priming Sites Eliminate the Major Class of False Positive Colonies during Negative Selection-Mediated Recombineering

**Figure 2:**
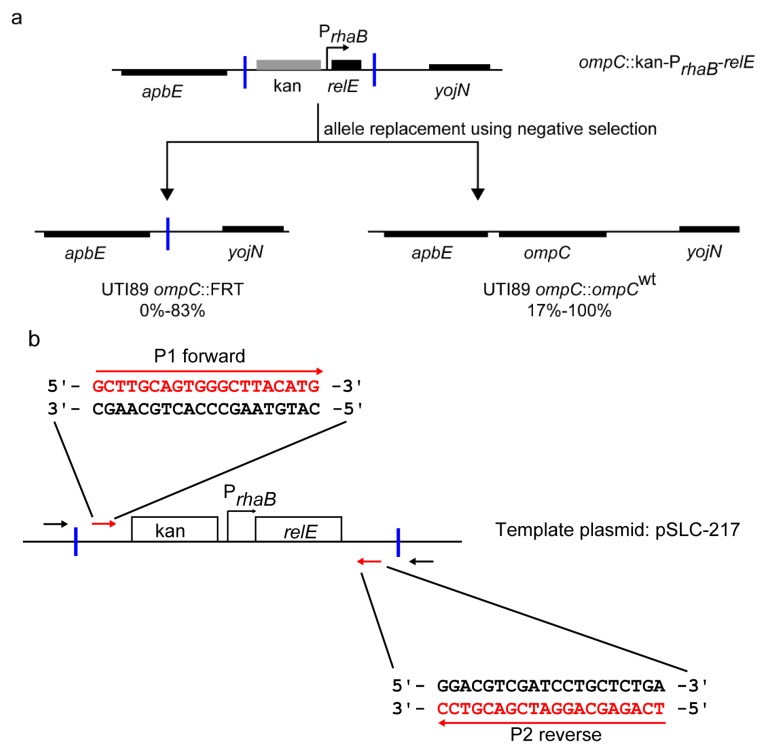
Elimination of undesired FRT recombinants during recombineering. (**a**) Genetic organization of the major recombinant products found during recombineering. The *ompC* locus and surrounding genes are shown as an example. Top, genetic organization of the initial *ompC* knockout strain (*ompC*::kan-P_*rhaB*_-*relE*) created by recombineering using positive selection for kanamycin. Bottom left, genetic organization of the product of undesired recombination between FRT sites during subsequent allele replacement by recombineering using negative selection (*ompC*::FRT). Bottom right, the genetic organization of the desired recombination where a restored wt *ompC* allele has replaced the positive-negative selection cassette during recombineering using negative selection (*ompC*::*ompC*^wt^). The percentage of colonies in each class, based on PCR screening after negative selection, is shown below each diagram (data taken from 30 recombineering steps using negative selection in 4 loci in UTI89). Blue vertical bars indicate FRT sites. (**b**) Redesigned priming sites for template plasmid pSLC-217 (and other pKD4 derivatives). Red arrows indicate new priming sites which exclude flanking FRT sites in the resultant amplified positive-negative selection cassette. New primer sequences are indicated in red as P1 forward and P2 reverse. Black arrows indicate the original priming sites 1 and 2 (for pKD4). Blue vertical bars indicate FRT sites. New primer sequences for pKD3 derivatives are listed in Table S3.

All *fimH* allelic replacements described above were made using the kan-P_*rhaB*_-*relE* cassette amplified from template plasmid pSLC-217, which is derived from pKD4 [20]. We used priming sites 1 and 2, originally recommended for pKD4, which resulted in inclusion of Flp flippase recognition target (FRT) sites flanking the selection cassette [20]. While creating allelic variants in *fimH* and other loci (such as *ompC*) in UTI89, we observed variable numbers of false-positive background colonies during the final negative selection recombineering step (0%–83%). While these are screened out by routine verification of isolated colonies, further examination revealed that recombination at the flanking FRT sites (leading to elimination of the cassette equivalent to “Flp-ing” it out) was a common mechanism for these undesired background colonies (Figure 2a). We therefore designed alternative priming sites (primers; P1 forward, P2 reverse, Table S3) for the template plasmids that excluded the FRT sites (Figure 2b); as expected, this eliminated this class of background colonies in negative selection-mediated allele replacements. This increased the efficiency of the negative selection step to nearly 100% (nearly all colonies growing have the intended recombination event), simplifying subsequent strain verification, although this eliminates the possibility of using Flp recombinase to eliminate the positive-negative selection step after the initial gene knockout.

## 3. Experimental Section

### 3.1. Bacterial Strains and Plasmids

Bacterial strains and plasmids used in this study are listed in Table S1.

### 3.2. Chromosomal FimH Mutations and Recombineering

SLC-502 (UTI89 *fimH*::kan-P_*rhaB*_-*relE*) was used for all allelic replacements. Previously reported marked *fimH* mutants were used as templates to amplify *fimH* alleles [14] using primers UTI89+4913234 *fimH* and UTI89-4914539 *fimH* (Table S3). Recombineering was performed as previously described [19].

### 3.3. PCR and Sequencing

Colony PCRs were used to check for insertion/replacement of the selection cassette with locus-specific primers (Table S3). All mutations were confirmed by using Sanger sequencing (1st base, Singapore) on PCR products with the same primers used for amplification.

### 3.4. Hemagglutination Assay

These were performed as previously described [14].

### 3.5. In Vivo Mouse Infections

All bacterial strains were cultured in type 1 pili-inducing conditions and used to infect seven- to eight-week-old C3H/HeN female mice (InVivos, Singapore) as previously described [21].

## 4. Conclusions

Using a large series of scarless, unmarked FimH mutants, we have recapitulated previously reported FimH phenotypes, validating the assignment of them to FimH mutations. This further demonstrates that using a combined positive-negative selection cassette is an effective way to generate definitive genetic constructs in UTI89. We have further improved the efficiency of the negative selection step by eliminating one class of undesired recombinations. These protocols and cloning strategies can also be used to generate definitive genetic constructs in other UPEC, *E. coli*, and other Enterobacteriaceae.

## Supplementary Materials

The following are available online at www.mdpi.com/2076-0817/5/1/9/s1, Table S1: Strains and plasmids used in this study, Table S2: FimH mutations and HA titers in UTI89, Table S3: Primers used in this study.
