# Supplementary material for: Application and Optimization of relE as a Negative Selection Marker for Making Definitive Genetic Constructs in Uropathogenic Escherichia coli
Source: Pathogens. 2016 Jan 18;5(1):9. doi: 10.3390/pathogens5010009 (PMC4810130; doi:10.3390/pathogens5010009)
Supplement: Supplementary file 1 [file pathogens-05-00009-s001.pdf]

Supplementary Materials: Application and Optimization of *relE* as a Negative Selection Marker for Making Definitive Genetic Constructs in Uropathogenic *Escherichia coli*

Varnica Khetrapal, Kurosh S. Mehershahi, Siyi Chen and Swaine L. Chen

Table S1. Strains and plasmids used in this study.

Strain	Genotype	Source/Reference
SLC-6	Wild type UTI89	Chen *et al.*, 2006 [1]
SLC2-33-1	UTI89 *fimH*::*fimH*^{UTI89}-kan	Chen *et al.*, 2009 [2]
SLC2-46-1	UTI89 *fimH*::*fimH*^{A62S}-kan	Chen *et al.*, 2009 [2]
SLC2-51-1-1	UTI89 *fimH*::*fimH*$^{A27V/V163A}$-kan	Chen *et al.*, 2009 [2]
SLC2-35-1	UTI89 *fimH*::*fimH*^{Q133K}-kan	Chen *et al.*, 2009 [2]
SLC2-68-4-1	UTI89 *fimH*::*fimH*$^{A27V/A62S/S70N/N78S}$-kan	Chen *et al.*, 2009 [2]
SLC2-39-1	UTI89 *fimH*::*fimH*^{A27V}-kan	Chen *et al.*, 2009 [2]
SLC2-45-4	UTI89 *fimH*::*fimH*^{V163A}-kan	Chen *et al.*, 2009 [2]
SLC2-89-3	UTI89 *fimH*::*fimH*$^{A27V/A62S/V163A}$-kan	Chen *et al.*, 2009 [2]
SLC-502	UTI89 *fimH*::kan-P$_{rhaB}$-*relE*	Khetrapal *et al.*, 2015 [3]
SLC-463	UTI89 *fimH*::*fimH*^{UTI89}	Khetrapal *et al.*, 2015 [3]
SLC-464	UTI89 *fimH*::*fimH*^{A62S}	Khetrapal *et al.*, 2015 [3]
SLC-465	UTI89 *fimH*::*fimH*$^{A27V/V163A}$	Khetrapal *et al.*, 2015 [3]
SLC-478	UTI89 *fimH*::*fimH*^{Q133K}	Khetrapal *et al.*, 2015 [3]
SLC-479	UTI89 *fimH*::*fimH*$^{A27V/A62S/S70N/N78S}$	This study
SLC-482	UTI89 *fimH*::*fimH*^{A27V}	This study
SLC-483	UTI89 *fimH*::*fimH*^{V163A}	This study
SLC-484	UTI89 *fimH*::*fimH*$^{A27V/A62S/V163A}$	This study
SLC-516	UTI89 *ompC*::kan-P$_{rhaB}$-*relE*	Khetrapal *et al.*, 2015 [3]
Plasmid	Genotype	Source/Reference
pSLC-217	kan-P$_{rhaB}$-*relE*	Khetrapal *et al.*, 2015 [3]
pKM208		Murphy and Campellone, 2003 [4]

Table S2. FimH mutations and HA titres in UTI89.

Genotype	Hemagglutination Assay Titres	
	Using Positive Selection (Residual Kanamycin Marker)	Using Negative Selection (No Selection Marker)
Wild type UTI89	8	8
UTI89 *fimH*::*fimH*^{UTI89}	8	8
UTI89 *fimH*::*fimH*^{A62S}	5	5
UTI89 *fimH*::*fimH*$^{A27V/V163A}$	7	7
UTI89 *fimH*::*fimH*^{Q133K}	4	4
UTI89 *fimH*::*fimH*$^{A27V/A62S/S70N/N78S}$	8	8
UTI89 *fimH*::*fimH*^{A27V}	8	8
UTI89 *fimH*::*fimH*^{V163A}	8	8
UTI89 *fimH*::*fimH*$^{A27V/A62S/V163A}$	8	8

Table S3. Primers used in this study.

No	Sequence	Notes
1	AGTGATTAGCATCACCTATACCTACAGCTGAACCCAAAGAGATGAT TGTAGTGTAGGCTGGAGCTGCTTC	*fimH* knockout Forward
2	TAGCTTCAGGTAATATTGCGTACCTGCATTAGCAATGCCCTGTGATT TCTCATATGAATATCCTCCTTAG	*fimH* knockout Reverse
3	ATGATGTTGCGCTTGAGTTG	UTI89+4913234 *fimH*
4	ATCTGGCCTACAAAGGGCTAA	UTI89-4914539 *fimH*
5	ATACCCCTTAATGCCCCATC	UTI89+4912861 test *fimH* KO
6	ACCGCGCAAAACATCCAGTT	UTI89+4913338 test *fimH* KO
7	GGTATTCGGCATTGGCCTGA	UTI89-4914820 test *fimH* KO
8	GCTTGCAGTGGGCTTACATG	P1 forward for amplification of dual selection cassette with kanamycin without FRT sites (from template plasmid pSLC-217 and related plasmids (Khetrapal *et al.*, 2015 [3])
9	TCAGAGCAGGATCGACGTCC	P2 reverse for amplification of dual selection cassette with kanamycin without FRT sites (from template plasmid pSLC-217 and related plasmids (Khetrapal *et al.*, 2015 [3]))
10	CTTCATTTAAATGGCGCGCC	P3 forward for amplification of dual selection cassette with chloramphenicol without FRT sites (from template plasmid pSLC-242 and related plasmids (Khetrapal *et al.*, 2015 [3]))
11	CATATGAATATCCTCCTTAG	Datsenko and Wanner original primer at priming site 2 (Datsenko and Wanner 2000 [5]). For amplification of dual selection cassette with chloramphenicol without FRT sites (from template plasmid pSLC-242 and related plasmids (Khetrapal *et al.*, 2015 [3]))

References

1. Chen, S.L.; Hung, C.S.; Xu, J.; Reigstad, C.S.; Magrini, V.; Sabo, A.; Blasiar, D.; Bieri, T.; Meyer, R.R.; Ozersky, P.; et al. Identification of genes subject to positive selection in uropathogenic strains of Escherichia coli: A comparative genomics approach. Proc. Natl. Acad. Sci. USA 2006, 103, 5977–5982.
2. Chen, S.L.; Hung, C.S.; Pinkner, J.S.; Walker, J.N.; Cusumano, C.K.; Li, Z.; Bouckaert, J.; Gordon, J.I.; Hultgren, S.J. Positive selection identifies an in vivo role for FimH during urinary tract infection in addition to mannose binding. Proc. Natl. Acad. Sci. USA 2009, 106, 22439–22444.
3. Khetrapal, V.; Mehershahi, K.; Rafee, S.; Chen, S.; Lim, C.L.; Chen, S.L. A set of powerful negative selection systems for unmodified Enterobacteriaceae. Nucl. Acids Res. 2015, doi:10.1093/nar/gkv248.
4. Murphy, K.C.; Campellone, K.G. Lambda Red-mediated recombinogenic engineering of enterohemorrhagic and enteropathogenic E. coli. BMC Mol. Biol. 2003, 4, 11.
5. Datsenko, K.A.; Wanner, B.L. One-step inactivation of chromosomal genes in Escherichia coli K-12 using PCR products. Proc. Natl. Acad. Sci. USA 2000, 97, 6640–6645.
